# Reshaping the Concept of Riedel’s Thyroiditis into the Larger Frame of IgG4-Related Disease (Spectrum of IgG4-Related Thyroid Disease)

**DOI:** 10.3390/biomedicines11061691

**Published:** 2023-06-11

**Authors:** Mara Carsote, Claudiu Nistor

**Affiliations:** 1 Department of Endocrinology, Carol Davila University of Medicine and Pharmacy & C.I. Parhon National Institute of Endocrinology, 011863 Bucharest, Romania; 2 Department 4—Cardio-Thoracic Pathology, Thoracic Surgery II Discipline, Carol Davila University of Medicine and Pharmacy & Thoracic Surgery Department, Dr. Carol Davila Central Emergency University Military Hospital, 050474 Bucharest, Romania; ncd58@yahoo.com

**Keywords:** Riedel’s thyroiditis, IgG4-related disease, thyroidectomy, thyroid, autoimmune, immunoglobulin, IgG4, IgG4-related thyroid disease, antibodies, immunohistochemistry

## Abstract

Recently, Riedel’s thyroiditis (RT) was assimilated into the larger spectrum of immunoglobulin IgG4-related disease (IgG4-RD) in addition to a particular frame of IgG4-related thyroid disease (IgG4-RTD), underlying IgG4-RT, IgG4-associated Hashimoto’s thyroiditis (and its fibrotic variant), and IgG4-related Graves’s disease. Our objective was to overview recent data on RT, particularly IgG4-RD and IgG4-RTD. The case and study– sample analysis (2019–2023) included 293 articles and selected 18 original studies: nine single case reports (N = 9, female/male = 2/1, aged: 34–79 years, 5/9 patients with serum IgG4 available data, 2/5 with high serum IgG4) and four case series (N = 21; 4/5 series provided data on IgG4 profile, 3/21 had serum IgG4 assays, and 2/3 had abnormally high values). IgG4-RD and thyroid findings were analyzed in three cohorts (N = 25). Another two studies (N = 11) specifically addressed IgG4-RTD components. On presentation, the patients may have hypothyroidism, transitory thyrotoxicosis, goiter, long-term history of positive anti-thyroid antibodies, and hypoechoic ultrasound thyroid pattern. The 5-year analysis (N = 66) showed the rate of serum IgG4 evaluation remained low; normal values do not exclude RT. Mandatory histological and immunohistochemistry reports point out a high content of IgG4-carrying plasma cells and IgG4/IgG ratio. Unless clinically evident, histological confirmation provides a prompt indication of starting corticoid therapy since this is the first-line option. Surgery, if feasible, is selective (non-responders to medical therapy, emergency tracheal intervention, and open/core needle biopsy). Current open issues are identifying the role of serum IgG4 assays in patients with IgG4-RD, finding out if all cases of RT are IgG4-mediated, applying IgG4-RTD criteria of differentiation among four entities, and providing an RT/IgG4-RTD guideline from diagnosis to therapy. It remains that the central aim of approaching RT in daily practice is the early index of suspicion in order to select patients referred for further procedures that provide enough histological/immunohistochemistry material to confirm RT and its high IgG4 burden.

## 1. Introduction

**Figure 1:**
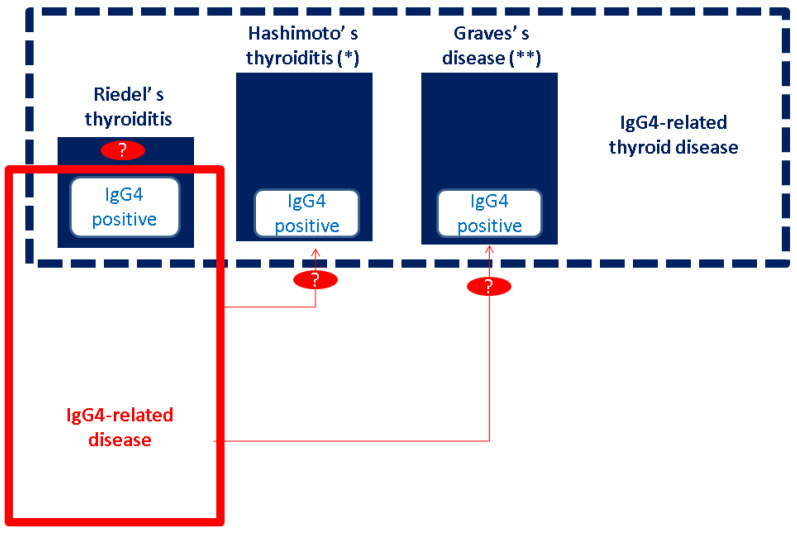
The current concept of RT: IgG4-related thyroid disease includes IgG4-mediated RT, Hashimoto’s thyroiditis and its fibrotic variant, and Graves’s disease (including some cases complicated with thyroid eye disease); RT has a higher risk of involving other (non-thyroid) sites, specified as IgG4-related disease [1,2,3]. Abbreviations: Ig = immunoglobulin; * fibrotic variant of Hashimoto’s thyroiditis; ** thyroid eye disease; “?” at RT means we currently do not have enough information to specify if all cases of RT are IgG4-mediated; and “?” for red arrows means that we currently do not have data to connect IgG4-RD with Hashimoto’s thyroiditis and Graves’s disease to the degree that it has been reported in RT (of note, the two different sizes of blue boxes in the figure suggest that RT has a less epidemiological impact as opposed to the other two entities in IgG4-RTD).

Riedel’s thyroiditis (RT), being strongly connected with positive antibodies against the thyroid, has been traditionally regarded as a particular type of autoimmune thyroiditis. However, during the last decade, RT was assimilated into the larger spectrum of immunoglobulin IgG4-related disease (IgG4-RD) and, recently (in 2021), a proposal was released to designate a particular framework, namely, IgG4-related thyroid disease (IgG4-RTD) with four underlying entities: IgG4-mediated RT, IgG4-associated Hashimoto’s thyroiditis (and its fibrotic variant), and IgG4-related Graves’s disease [1,2,3] (Figure 1).

### 1.1. Riedel’s Thyroiditis: Classical Approach

The classical concept of RT involves a chronic inflammatory condition with a predominantly fibrotic pattern of infiltration located at the thyroid and surrounding areas (parathyroid glands, muscles, trachea, esophagus, local nerves, and vessels). The level of statistical evidence is low (mostly of case reports and series); an estimated incidence of 1 to 1.06 cases per 100,000 people has been estimated, with women being more often affected (female to male ratio of 3–5 to 1), particularly those aged between 30 and 50 years [2,4,5].

Riedel’s thyroiditis results in a thyroid gland that is mostly firm (“woody”, “stony”, or “iron-hard”) and enlarged causing local pain, compressive symptoms (dyspnea, dysphagia, hoarse voice, respiratory insufficiency), and hypothyroidism (up to 80% of cases) in association with extra-thyroid fibrosis; unusual complications such as exophthalmos, Horner’s syndrome, or venous sinus thrombosis have been found as well [2,4,6].

Positive serum antibodies such as anti-thyroid peroxidase and anti-thyroglobulin are identified in 9 out of 10 patients. Blood inflammatory markers such as elevated C-reactive protein (CRP) or erythrocyte sedimentation rate (ESR) may be present in typical presentations associated with full-blown clinical manifestations [2,4,7].

Ultrasound and computed tomography might help the imaging diagnosis, particularly to prove the hypoechoic ultrasound pattern in association with avascular gland enlargement at Doppler examination and to show thyroid and extra-thyroid fibrosis extension at computed tomography or magnetic resonance imaging. Furthermore, ultrasound and computed tomography are also used to differentiate RT from malignancy (which is not always feasible unless histological evidence is provided) and to reveal non-thyroid spreading of IgG4-RD. Ultrasound elastography might highlight an elevated stiffness. 18-Fluoro-deoxyglucose positronic emission tomography/computed tomography may point out an increased tracer uptake at first presentation, while technetium (Tc) thyroid uptake is reduced at 99mTc thyroid scintigraphy [2,8,9].

RT confirmation comes from histological reports (in addition to immunohistochemistry analysis) based on lymphocyte infiltration, fibrosis, and destruction of thyroid follicles (which explains the long-standing primary hypothyroidism). No malignant or giant cell should be identified. A high amount of IgG4-carrying plasma cells represents the novel clue of RT. Of course, in order to provide the histological and immunohistochemistry features, an open/core needle biopsy is necessary (unless a thyroidectomy was already performed); thus, an index of suspicion should be kept in mind even in the early stages of the disease when classical clinical signs are not yet very suggestive [2,6,10].

Misdiagnosis as Hashimoto’s thyroiditis (mostly due to positive serum antibodies against the thyroid) or as a thyroid malignancy, particularly of anaplastic carcinoma, sarcoma, or primary thyroid lymphoma, is often described considering the anatomical aspects and progressive (severe) clinical evolution [2,11]. Delay of the diagnosis underlines a time window from presentation to histological confirmation varying between a few months and 2 years (a median of 4 months) [2,6].

Prompt intervention might improve the outcome. There is no consensus therapy in RT, which is rather a matter of individual decision, but glucocorticoid treatment (for instance, with prednisone, prednisolone, or dexamethasone) represents the first choice of medication. Currently, rituximab, a monoclonal antibody against CD20 protein, may be regarded as a second-line therapy. Tamoxifen (10–20 mg twice per day) has a longer history of use than rituximab as an additional drug to corticoids; it may be added to them or even offered as a single medication. This selective estrogen receptor modulator is beneficial not through its anti-estrogen capacity but via anti-fibrotic effects by stimulating transforming growth factor (TGF-*β*), a cytokine released by fibroblasts and epithelial cells acting as a growth inhibitor of various cells (including fibroblasts) [12,13]. The need for anti-fibrotic effects also indicates mycophenolate mofetil (1 g twice per day) which is approved for systemic fibrosis, but it has limited applications due to severe side effects (pancytopenia or renal function damage) [2,6,14].

Low-dose radiation therapy at the thyroid level represents a rarely applied alternative [15]. Surgery, despite not being a first choice of therapy, is useful for surgical biopsy (since fine needle aspiration is not helpful in more than 75% of cases), for refractory cases to medical therapy, or for emergency tracheostomy [2,4,6]. Generally, the presence of fibrosis and invasion of surrounding areas increases the rate of post-thyroidectomy complications or failure to completely remove the thyroid gland [16,17,18]. However, in cases of RT, it helps to prove a pathological confirmation. On the contrary, a patient who is mistakenly diagnosed with RT and actually has an alternative diagnosis may benefit from a post-operatory histological report [19,20].

The natural history of RT is progression but spontaneous regression or episodes of relapse are reported in addition to medically-induced remission, which is mostly due to glucocorticoids. Disease-related mortality is rather low, but a severe impairment of quality of life is reported due to multiple complications and long-term medication [2,6].

Novel practical approaches to RT connect the disease with the larger area of IgG4-RD in terms of serum IgG4 assays but, mostly, the intra-thyroid IgG4 findings reflect their essential role in RT. The importance of serum IgG4 assays has been suggested for daily practice purposes and further guidelines are required. Controversies related to their low predictive value and correlations with active or inactive stages of the condition are still ongoing. Immunohistochemistry-based IgG4 stain is mandatory for RT confirmation by pointing out a high amount of IgG4-carrying plasma cells, and an increased IgG4/IgG ratio [21].

### 1.2. Immunoglobulin G4-Related Disease

IgG4-RD, a rare immunologic condition, presents as single or multi-organ/tissue spreading at pulmonary, orbital areas, salivary glands (such as chronic sclerosing sialadenitis, also named Kűttner tumor, and Mikulicz’s disease), biliary ducts and in the gallbladder (sclerosing cholangitis and cholecystitis), renal system (interstitial nephritis), pancreatic system (multifocal autoimmune pancreatitis), and lymph nodes in association with various cardiovascular manifestations (such as aortitis, pericarditis, vasculitis, and pulmonary vascular disease) [22,23,24,25,26]. Virtually, any organ may become a host of the condition [27]. This non-malignant, fibro-inflammatory disease requires a multidisciplinary team since it involves a heterogeneous presentation, thus awareness is essential despite a rather low epidemiologic impact (it is currently being considered an orphan disease) [28,29].

An IgG4-associated entity was first reported more than two decades ago (in 2001) in terms of pancreatitis (and further conceptual data were soon published in 2003) [30,31]; however, overall IgG4 dynamics massively changed over the years, with RT being recognized as part of this spectrum only one decade later and included in the IgG4-RD guideline from 2019 [32,33,34].

At the onset, the lesions might mimic a malignancy (or a paraneoplastic syndrome) or an infectious disease or they may be mistaken as another autoimmune condition, while severe cardiac and vessel involvement should be differentiated from an acute myocardial infarction or rupture of an aortic aneurysm [25,29,35]. Functional imaging with 18-Fluoro-deoxyglucose positronic emission tomography/computed tomography, despite not being specific, represents a useful tool to indicate the sites of IgG4-mediated increased activity in addition to traditional ultrasound, computed tomography, and magnetic resonance imaging-based findings [36,37].

The pathogenic traits of RT are yet poorly understood and are mostly related to polyclonal activation of plasma cells [28]. The serologic hallmark is represented by the elevated IgG4 subtype [22]. Genetic susceptibility regarding *CFHR*1 and *CFHR*4 gene deletions has been suggested [25,38]. The highly specific histologic features include an increased (dense) lymphoplasmacytic infiltration, high content of IgG4-positive plasma cells, storiform fibrosis, and (obliterate) phlebitis [22,24]. Needle biopsy is less likely to point out all these mentioned traits compared with surgical (open) biopsy depending on the organ and disease severity [39].

In terms of treatment, firstly, patients are candidates for glucocorticoid therapy, which might rapidly improve the clinical evolution in the majority of cases; thus, prompt and adequate disease recognition and intervention is important (if feasible) [24]. Non-responders are offered second-line medication such as rituximab, as well as other drugs such as cyclophosphamide or mycophenolate mofetil, etc. [22,28,40]. Surgical interventions are required in selected cases, typically after the failure of medical management. Sometimes, interventions include emergency procedures as seen in vascular complications (for instance, valve replacements) [41,42,43].

### 1.3. Aim

Our objective is to review recent data on RT, particularly concerning IgG4-RD and IgG4-RTD.

## 2. Materials and Methods

This is a narrative review. The case and study sample analysis included articles according to the following inclusion and exclusion criteria as displayed in Table 1.

## 3. Results

**Figure 2:**
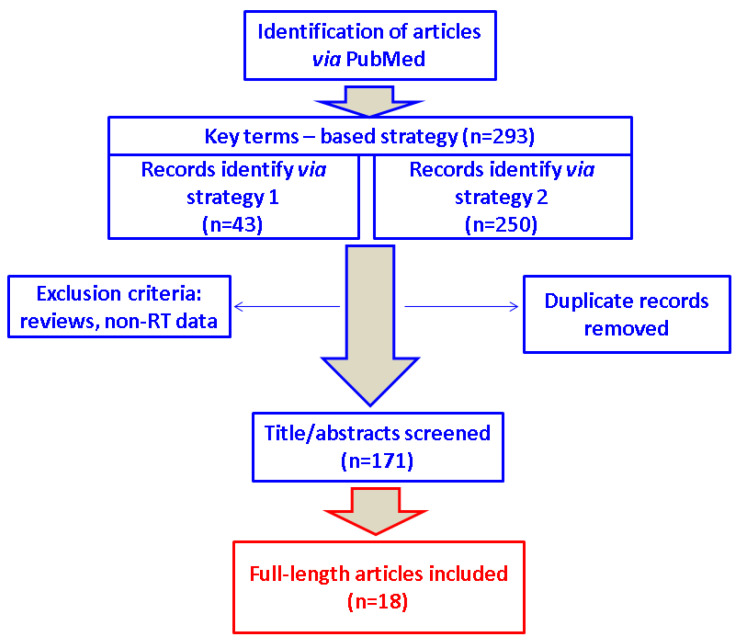
Flow diagram of research according to the mentioned strategy. Abbreviations: RT = Riedel’s thyroiditis; n = number of papers.

We identified 293 articles (43 according to strategy 1, and 250 through strategy 2) and selected 18 original papers for the final analysis (nine case reports, four case series, three studies on IgG4-RD, and two studies on IgG4-RTD) (Figure 2).

### 3.1. Riedel’s Thyroiditis: Presentation

Since 2019, nine case reports of a single patient per paper have been published according to our methods [6,44,45,46,47,48,49,50,51] (Table 2).

On admission, a patient (who is eventually confirmed with RT) may have a long history of multinodular goiter, Hashimoto’s thyroiditis (in terms of elevated serum positive antibodies against thyroid according to usual diagnosis in daily practice), or hypoechoic pattern at ultrasound with or without associated hypothyroidism and not being categorized as RT during this time period unless a sudden/progressive thyroid enlargement with compression effects emerges [6,44]. Gökçay Canpolat et al. [52] reported that only 25% of their series had positive anti-thyroid antibodies, suggesting that not all individuals display serological confirmation of Hashimoto’s thyroiditis. This was a case series of eight patients (between 2000 and 2019) with a mean age of 40.5 ± 6.8 years (seven females and one male), representing a lower percentage of positive antibodies than expected according to other published data [52]. Interestingly, Jin et al. [53] identified a 72-year-old male with flare-up (self-remitted) thyrotoxicosis at the onset, which is not the norm, as opposed to traditional thyroiditis-associated thyrotoxicosis in chronic autoimmune and subacute types [53]. A history of goiter might even include a prior partial resection; for example, one adult female had a partial thyroidectomy completed 12 years before the current episode of RT [46].

Hypoparathyroidism due to RT-associated extensive fibrosis is found at first presentation, as well as a surgically-induced form in cases that require a large dissection or display adherent lesions at cervical areas [53]. For instance, Salhi et al. [44] reported an adult female in her late 40s admitted for RT after a long evolution of goiter with hypothyroidism; she was diagnosed with transitory hypoparathyroidism at first RT presentation which remitted after a 1-year therapy with prednisone (20 mg/day) [44]. Shafi et al. [49] described a male case with RT who was already under therapy with calcium and alfacalcidol in addition to levothyroxine when the final recognition of RT was conducted [49]. A transitory case of hypoparathyroidism was reported by Er-Rahali et al. [45] after total thyroidectomy was converted to a subtotal procedure due to stony thyroid consistency followed by a post-operatory disease progression requiring glucocorticoid intervention [45].

### 3.2. Riedel’s Thyroiditis: Management and Outcome

The modern approach to RT, if suspected, takes into consideration the IgG4 profile. Serum IgG4 might not be increased in local (thyroid) forms of RT (with no other spreading of IgG4-RD) while immunohistochemistry provides a meticulous insight into IgG4-positive staining, which provides a clue regarding the specific case (regardless of the concentration of the serum IgG4 profile) [45]. Five out of the nine single case reports had a serum IgG4 assay available (only 2/5 had higher than normal values), while all subjects had a histological confirmation of the condition [6,44,45,46,47,48,49,50,51].

Glucocorticoid therapy improves the clinical presentation; if the onset is severe due to local compressive symptoms, prompt intravenous intervention is required (for example, prednisolone 2–3 mg/kg/day, 2–7 days) [52]. In less severe cases, the oral route may be used from the beginning, for example, prednisolone 0.8–1 mg/kg/day, 4–8 weeks, as reported by Gökçay Canpolat et al. [52]. Similarly, prednisolone 60 mg/day (1 mg/kg/day) was reported to associate with clinical improvement, and ultrasound assessment showed a mild volume reduction after 6 weeks as reported by Góralska et al. [46]. Long-term medication with oral glucocorticoids depends on the clinical outcome and the decrease in gland enlargement; it may be typically prolonged to one year [52]. Of note, Shafi et al. [49] treated their 35-year-old patient with prednisolone 5 mg per day for 2 years. The patient developed thrombosis under tamoxifen (requiring long-term oral anticoagulants) and did not tolerate rituximab [49].

Gökçay Canpolat et al. [52] added tamoxifen as a second-line therapy while the patients were under oral corticoid medication for 1 to 3 months to enhance the effects of glucocorticoids before stopping them [52]. Navarro-Sánchez et al. [48] described the case of a 69-year-old female who was treated only with tamoxifen (20 mg per day for 2 months) in addition to levothyroxine replacement (100 µg per day) for hypothyroidism and registered an excellent response [48]. Of course, all the subjects admitted with a history of hypothyroidism, with RT-induced hypothyroidism, or those who developed post-thyroidectomy hypothyroidism received lifelong levothyroxine replacement [6,44,45,46,47,48,49,50,51].

Mammen et al. [50] revealed the third ever RT patient treated with rituximab in 2019 (after prior reports were completed in 2013 and 2018, respectively) [53,54,55,56]. This was a 51-year-old woman who became unresponsive to glucocorticoids and tamoxifen after a few months; thus, intravenous rituximab was initiated for four doses with a good response (which allowed stopping tamoxifen and reduction in the prednisone dose) [50].

A thyroidectomy, even if unsuccessful when performing a complete gland resection, serves two essential purposes: one is placing a transitory lifesaving tracheostomy if needed due to tracheal fibrosis and inflammation and the other is providing histological material to point out the IgG4-associated tissue burden [45]. Despite the failure of complete thyroid removal, intra-operatory biopsy allowed the recognition of RT in order to immediately start glucocorticoid therapy. Massive local fibrosis is prone to post-surgery complications [45,49,50]. For instance, Pandev et al. [6] identified a 34-year-old female with RT diagnosis after a 2-year history of Hashimoto’s thyroiditis (according to her high serum antibodies). After that, the severe local evolution required an emergency intervention with a tracheotomy. The open biopsy provided enough histological evidence to confirm RT and to initiate corticoid therapy. Surgical correction of the post-tracheotomy tracheal-cutaneous fistula was necessary during the surveillance period, suggesting the need for a good collaborative team decision in this situation [6]. Successful total thyroidectomy has been reported in some cases [54,57], while in others, the patient refused the intervention [46].

Generally, in endocrine practice, fine needle aspiration is the first choice in different lesions at the thyroid after initial clinical and ultrasound detection regardless of the underlying pathological report [58,59]. Yet, in RT the procedure is not useful for diagnosis as pointed out by our sample-based analysis (which in some cases was not feasible at all due to “iron-hard” thyroid consistency) [45,46,49,51]. Among the spectrum of IgG4-RTD, the fibrotic variant of Hashimoto’s thyroiditis has a certain similar cytological profile to RT. If surgery is not mandatory due to compressive features or it is not approved by the patient or contra-indicated, core needle biopsy represents a more practical alternative (if feasible) [51].

Histological analysis after biopsy and/or thyroidectomy in association with IgG4 immunostaining was applied in three out of the four case series we could identify (case series of more than one patient with RT per paper) [54,57,60] (Table 3).

Generally, IgG4-RD criteria established by the American College of Rheumatology (ACR) and the European League Against Rheumatism (EULAR) in 2019 took into consideration RT as part of “head and neck gland involvement”. Hashimoto’s thyroiditis may be incidental (distinct from IgG4-RD), but it is part of the RT-associated picture if RT represents the single organ manifestation. As seen in other sites, the positive diagnostic of IgG4-RD is established by scoring clinical and radiological organ-specific findings or highly suggestive parameters provided by the pathological report. Moreover, serum IgG4 assays might prove normal in RT; however, a level of more than 2–5 times above the normal limit (especially above five times) represents one criterion associated with different scores depending on serum levels. Exclusion criteria are required, too, for providing a positive diagnosis of IgG4-RD [32,33]. In 2021, Takeshima et al. [1] proposed a panel of specific criteria to navigate among IgG4-RTD in terms of diagnosis (not therapeutic approach, which remains an open issue) [1].

According to our sample-based analysis, we identified one study published in 2021 by Yu et al. [60] that was a retrospective series of five subjects with RT (80% females, aged between 33 and 56 years); IgG4 status has been assessed at the immunohistochemistry report in terms of IgG4-carrying plasma cells per high-power field (HPF) and IgG4/IgG4 ratio (the individuals were admitted as patients between 2000 and 2019, having a median follow-up of 7 years). The subjects (4/5) fulfilled the mentioned criteria [1] of having >10 IgG4-positive plasma cells/HPF and, also, displaying an IgG4/IgG ratio of at least 20% [60]. Of note, in this series, only two subjects had a ratio higher than 40%, a cut-off that has been previously proposed, but these findings suggest that a level of 20% is suitable for RT diagnosis as part of the Ig4-RD spectrum [1].

### 3.3. Patients with IgG4-Related Disease and Potential Thyroid Findings

Some studies enrolled patients with non-thyroid manifestations of IgG4-RD, but further on, the authors identified that subgroups of these subjects were associated with IgG4-mediated thyroid features consistent with IgG4-RTD. We identified three such studies [61,62,63] (Table 4).

One series of five subjects (mean age of 40.2 years) with IgG4-related vessel involvement at the aortic level and other arteries revealed a subject with RT (a diagnosis confirmed through thyroid biopsy). This was a 49-year-old female who presented complications at the carotid, subclavian, and pulmonary arteries, and a small increase in serum IgG4 without hypothyroidism. The general IgG4-related condition responded well to glucocorticoid therapy [61]. We also mention one case report of Pacella et al. [47] that identified a 53-year-old male with retroperitoneal fibrosis diagnosed 4 years after an episode of RT requiring decompression surgery at that moment, with both thyroid and retroperitoneal findings being elements of IgG4-RD [47]. Similarly, Azizi et al. [63] reported an experience with 12 subjects diagnosed with retroperitoneal fibrosis (mean age of 57 ± 10 years); among other specific complications and co-morbidities, one patient had RT [63].

## 4. Discussion

### 4.1. Practical Points on Riedel’s Thyroiditis

Currently, despite being an unusually rare entity, RT has been added to the larger register of IgG4-RD, thus many practitioners other than endocrinologists and surgeons might have to take into consideration thyroid involvement due to IgG4 connections [6,44,45,46,47,48,49,50,51,52,54,57,60,61,62,63]. Nowadays, it is essential to assess serum IgG4 levels but mostly to perform an immunohistochemistry exam in order to point out the IgG4 burden to have RT confirmation. Of course, many cases may remain underdiagnosed; thus, the level of awareness should be raised. RT-associated histological elements are post-operatory diagnosed in less than 0.05% of all thyroidectomies [4,54].

Another point is represented by the importance of the immediate use of glucocorticoids which remain the first-line approach. Other drugs such as tamoxifen and rituximab might prove useful in selected cases. Surgery, although not a first-line option unless an emergency, still represents a strong player in overall management from open biopsy to tracheal intervention [6,44,45,46,47,48,49,50,51,52,54,57,60,61,62,63]. Awareness of various issues is essential: from not being able to perform a complete thyroid removal due to massive fibrosis/stony consistency [44,54], to intra-operatory pneumothorax requiring thoracentesis, as seen in similar procedures for other etiological events [6,64,65]. Long-term complications such as tracheocutaneous fistulas require re-intervention [6].

A third chapter (also, an open chapter) is the potential role of other contributors to RT (other than IgG4). For instance, smoking might negatively influence an autoimmune disease as reported by Pandev et al. [6], but we have no convincing RT data so far. Positive family history of an autoimmune condition might be associated with a higher risk of developing RT. A family cluster of different autoimmune conditions seems important in RT according to Sun et al. [62]. The authors showed that among 628 patients with IgG4-RD, those with a positive family history of autoimmune disease (N′ = 93, representing 14.8%) had a younger age at IgG4-RD diagnostic, a higher rate of positive antinuclear antibodies, and an increased prevalence of RT as opposed to those with a negative family history (10.9% versus 2.4%, *p* = 0.001). Among endocrine conditions within the first subgroup, we mention Hashimoto’s thyroiditis in seven subjects (7.5%) and Graves’s disease in eight individuals (representing 8.6%) [62].

### 4.2. Non-RT Entities among the Spectrum of IgG4-Related Thyroid Disease

RT was first identified by the surgeon Bernhard Moritz Carl Ludwig Riedel in 1883 who later reported it in 1894 at a surgery conference and published it in 1896 [66]. Further on (in 1904), Hashimoto described fibrous thyroiditis, as well, and for a long period of time, it remained an open issue if the two conditions were actually the same [67,68]. The first case of IgG4-related Hashimoto’s thyroiditis was identified by evidencing a high content of IgG4-positive plasma cells (in 2009) [69]. Then, a fibrotic variant of this entity was described as part of the IgG4-RTD spectrum [21]. Dahlgren et al. [70] showed for the first time in 2010 that three patients with RT had an immunohistochemistry report consistent with a high burden of IgG4-carrying plasma cells [70].

**Figure 3:**
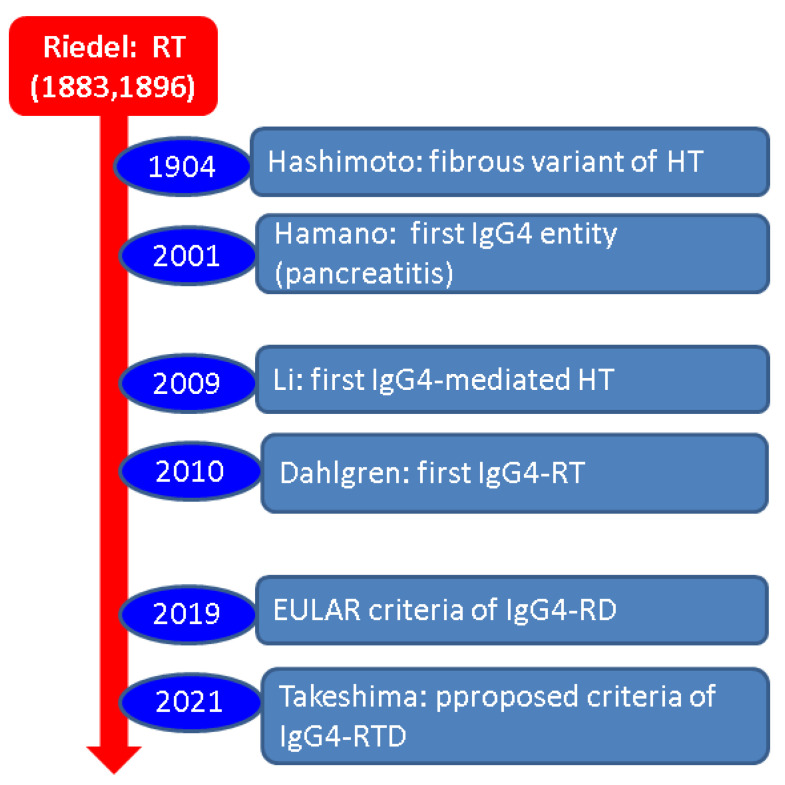
A timeline perspective of Riedel’s thyroiditis: considerations on IgG4 profile. Dr. Riedel first described Riedel’s thyroiditis (1883) and published it later (1896) [66,68]. Hashimoto described a fibrous variant of Hashimoto’s thyroiditis (1904) [68]. Hamano et al. described the first case of IgG4-associated disease (in the pancreas) in 2001 [68]. Li et al. discovered the first case of IgG4-associated Hashimoto’s thyroiditis in 2009 [69]. One year later, Dahlgren et al. identified the first three cases of IgG4-related RT [70]. The most recent guideline for IgG4-RD was released by EULAR in 2019, including data on the thyroid [32,33]. The proposed criteria for IgG4-RTD are published in 2021 [1]. Abbreviations: Ig = immunoglobulin; HT = Hashimoto’s thyroiditis; RT = Riedel’s thyroiditis; RD = related disease; and RTD = related thyroid disease.

In 2021, Takeshima et al. [1] proposed the nomenclature and the classification of IgG4-RTD since thyroid gland involvement, as opposed to other organs underlying IgG4-mediated co-morbidities, had not been a subject of specific diagnostic criteria/guidelines until two years ago. A combination of five elements in terms of serum IgG4, IgG4 histological content, thyroid enlargement, hypoechoic ultrasound pattern, and (non-thyroid) IgG4-RD allowed the stratification into “definitive, probable, or possible” IgG4-RTD. In any of the categories, clinical presentation (thyroid enlargement) and ultrasonography features (hypoechoic) are mandatory. Additionally, high IgG4 serum assays are required for a “definitive” diagnosis if histological evidence of elevated IgG4 content is present. The combination of clinical, ultrasound, and serum criteria allows only a “possible” diagnosis. Patients with IgG4-RD and clinical/ultrasound criteria have a “probable” IgG4-RTD. General (non-thyroid) spreading of the condition is not essential in order to obtain a positive diagnosis (if histological proof of thyroid involvement is provided) since a single-organ disease may be found [1] (Figure 3).

In 2021, Yu et al. [60] reviewed three databases concerning published cases of RT that provided enough information concerning positive IgG4 status. They identified 15 patients across 8 papers (13/15 patients had a thyroidectomy and 2/15 had an RT diagnosis based on core needle biopsy); 13/15 subjects had occlusive phlebitis according to the pathological exam; one individual had an extra-thyroid spreading; serum IgG4 assays were less frequently available (3/15); and half of these 15 persons had >10% IgG4-positive plasma cells/HPF and IgG4/IgG > 40% according to prior criteria for IgG4-RD [60,71]. As shown above, from 2019 to 2023, the number of published cases with an IgG4 profile in RT remained similarly low (please see Table 2, Table 3 and Table 4).

To the best of our knowledge, the highest number of RT-published cases so far is 212 (an article from 2020) [2]. Zala et al. [2] provided a systematic review/meta-analysis based on papers published from inception until September 2019 and accessed via PubMed/Medline and Web of Science. Demographic parameters included an average age at diagnosis of 47 years and a confirmation of female predominance (81%). The most frequent clinical elements were neck swelling (89%) followed by dyspnea in half of the cases and local pain in 41% of the patients. Lab findings showed that 70–97% of the individuals had positive inflammatory markers and less than half had positive antibodies against thyroid. Surgery was performed in 82% of the cases (total thyroid removal was carried out in one third of them). Corticoid therapy was used in 7 out of 10 patients (for a median of 3 months). Clinical improvement (to some extent) was registered in most of the cases. Overall, IgG4 profiling was not available in these cases, pointing out that the new era, namely, the IgG4 era in the RT approach, is yet to come [2].

IgG4-carrying cells (in addition to an increased IgG4/IgG ratio) are mandatory to be highlighted based on immunohistochemistry analyses. It is imperative to obtain enough tissue following thyroidectomy or open/core/surgical biopsy in order to analyze the IgG4 status. In addition to this assessment, meticulous histological description, clinical presentation (thyroid enlargement and woody consistency), and other fibrotic or sclerotic potential involvement at non-thyroid organs help the distinction among the four types of IgG4-RTD [72,73]. Some authors use the term “IgG4-related thyroiditis” mostly referring to IgG4-related Hashimoto’s thyroiditis (not RT). The clear distinction between these two entities is yet a matter of debate. Stony consistency seems to be the clinically distinctive feature of the fibrotic variant of Hashimoto’s thyroiditis [72,73]. Adams et al. [74] released some recommendations for reporting this subtype of IgG4-RTD: lack of extra-thyroid IgG4-RD, lack of disease spreading to thyroid surrounding tissues (which seems the usual signature of RT), stromal fibrosis (at least 30%) at the histological report, IgG4/IgG4 ratio of more than 30% while the required percent of IgG4-carrying plasma cells (per HPF) depends on stromal fibrosis distribution (>20/HPF for >50%, respective >30/HPF for 30–50%), and other pathological findings such as fibrosis at inter-follicular level and follicles destruction without phlebitis [74]. Another challenging differential diagnosis relates to IgG4-mediated Hashimoto’s thyroiditis and primary thyroid lymphoma [75]. On the contrary, Lintusaari et al. [76] identified a subgroup of individuals with IgG4-positive Hashimoto’s thyroiditis who displayed extra-glandular fibrosis [76].

Due to the rarity of IgG4-RTD, a limited number of clinical studies are reported so far, addressing distinct entities among the condition [53,77] (Table 5).

One study from Korea (between 2017 and 2021) included five patients with IgG4-RTD (two cases with IgG4-RT and three subjects with IgG4-mediated Hashimoto’s thyroiditis). The individuals with RT had an immunohistochemistry-based confirmation in terms of high content of IgG4-positive plasma cells and elevated IgG4/IgG ratio (42, 37%, respectively) [53]. Notably, Takeshima et al. proposed a cut-off criteria of >20 IgG4-plasma cells/HPF with a respective ratio of IgG4/IgG >30% in order to sustain the diagnosis of Ig4-RTD [1]. We also mention one study on 62 specimens after thyroidectomy that identified nine patients with RT. While IgG4 profiling was not available, other features such as positive stromal TTF-1, S100 protein (neuroectodermal cells), and lack of tumor markers such as CD56 and p63 were found to be distinctive in RT [77].

A subgroup of the patients confirmed with Graves’s disease (even complicated with eye involvement) might present an IgG4-related ophthalmic disease or they may associate other (non-thyroid) multi-organ manifestations of IgG4-RD [78,79]. A differentiation between eye involvement in IgG4-positive Basedow’s disease and IgG4-related ophthalmic disease as part of the IgG4-RD picture is mandatory [80]. The current medical approach to thyroid eye disease includes the understanding of new pathogenic traits and potential applications of new drugs based on immunomodulation and immunosuppression, including the loop of IgG4 [81,82,83,84,85]. For instance, one study from 2021 revealed on 69 consecutive subjects with Graves’ ophthalmopathy that one third of them had an increased level of serum IgG4 while blood IgG4 correlated with the disease’s activity and severity. However, this sub-group had a statistically significant better response (and outcome) to glucocorticoid therapy than patients with normal serum IgG4 [86]. Another study from 2023 on 60 patients with active Graves’ disease revealed that high serum IgG4 levels (which were found in 25% of the subjects) might serve as a marker of activity [81]. Luo et al. [87] suggested, according to their case-control study, that IgG4 assays also seem useful in follow-up assessments of subjects with ocular findings [87].

As mentioned, a small number of patients confirmed with Hashimoto’s thyroiditis develop its fibrotic variant, with this entity belonging to IgG4-RTD [88]. Most authors consider that the list of IgG4-RTD includes four distinct types of conditions, with Hashimoto’s thyroiditis being distinct from its fibrotic variant [88,89]. Some suggested that identifying a high level of serum IgG4 in one patient with Hashimoto’s thyroiditis represents an early predictor for developing its fibrotic variant [90]. Both, RT and the fibrotic variant of Hashimoto’s thyroiditis have been suggested to represent the end stage of a long progressive evolution, but typically a higher risk of non-thyroid IgG4-mediated fibrosis is reported in RT (as opposed to the other entity) [58,91].

Collaterally, we mention a different approach to serum IgG4 assays and thyroid lesions in a study conducted by Elshaer et al. [92]. The authors studied the predictive value of high IgG4 levels in patients with thyroid nodules that were assessed through fine needle aspiration based on cytological reports and found indeterminate results (N = 67 with Bethesda III and IV) and the subjects underwent total thyroidectomy (with a benign histological post-operatory report in 55 of them and malignancy in 12 subjects). The second subgroup had a statistically significant higher concentration of IgG4 (*p* = 0.0001), with the value of >180 mg/dL being associated with a 75% sensitivity and a 100% specificity. Serum antibodies against thyroid correlated with the levels of blood IgG4 (*p* = 0.001). This interesting approach requires further study. Currently, we do not place the serum assessment of IgG4 in daily endocrine practice as a prediction marker in suspected nodules for malignancy [92].

### 4.3. IgG4-RD: The Level of Thyroid Awareness

IgG4, a subclass of IgG, plays an important role in human immune response, particularly immune tolerance. The conditions involving this particular type of IgG4 are IgG4-autoimmune diseases or IgG4-RD characterized by fibrosis, sclerosis, and phlebitis [93]. We specifically choose to research the data starting from 2019 since the most recent criteria for IgG4-RD has been released, which raises IgG4 awareness with respect to thyroiditis [31,32]. Thyroid involvement in subjects with any location of an IgG4-RD might be expected in 20–40% of cases; however, according to older reports, it is only in 4% [94]. Clear evidence is still needed based on larger clinical trials. Similarly, there is a lack of data in pointing out if a subgroup of individuals diagnosed with an IgG4-associated RT is at higher risk to further develop IgG4-associated non-endocrine complications (grossly, one third of the patients with RT have fibrosis somewhere else in the body at some point in life) [47]. The thyroid gland findings may be concomitant to other sites or a patient already known to have an IgG4-mediated condition [31,32].

We cited Pacella et al. [47] reporting a male case of RT and retroperitoneal fibrosis, also belonging to the IgG4-RD, as well as the study of Azizi et al. [63] from 2020 on individuals with the same urologic condition [47,63]. Remarkably, this is an uncommon inflammatory finding consisting of fibrotic deposits at retroperitoneal space at the level of lumbar vertebras L2-5 [95]. The true epidemiologic profile remains unknown. While being asymptomatic for many years, it progressively expands causing local pain as the main element of presentation. The condition has been reported to be associated with other autoimmune conditions (apart from IgG4-RD), such as psoriasis and Hashimoto’s thyroiditis; however, the pathogenic connections remain poorly understood [96,97]. Contrast-enhanced computed tomography and magnetic resonance imaging help the diagnosis. The therapeutic approach includes glucocorticoid therapy, selective estrogen receptor modulators, such as tamoxifen (such as in RT), and immunosuppressant drugs, such as azathioprine, cyclophosphamide, cyclosporine, and mycophenolate mofetil. Subjects with poor responses to these are selectively referred to surgery [98,99].

Remarkably, a previously published study by Watanabe et al. [100] highlighted the thyroid involvement in 114 subjects diagnosed with IgG4-RD of various presentations (N1 = 92 with autoimmune pancreatitis; N2 = 15 with Mikulicz’s disease; and N3 = 7 with Ig4-associated cholangitis) and identified 22 of them (representing 19%) with hypothyroidism of any degree (half of them with clinically manifested hypothyroidism and the other half with a subclinical). The individuals with hypothyroidism had higher circulating levels of IgG, IgG4, circulating immune complex, beta2-microglobulin and lower C3, and increased thyroid volume versus patients with normal thyroid function (N″ = 92). Prednisone therapy improved the thyroid hormonal imbalance. Another study suggested that thyroid findings in patients with IgG4-RD might be based on a heterogeneous spectrum [100]. Perhaps, RT represents an extreme manifestation of this spectrum, which is, fortunately, rare enough.

IgG4-mediated endocrine diseases are IgG4-related thyroiditis of four types, as mentioned above, and IgG4-associated hypophysitis (which has been clarified within the last decade, but it is still a matter of debate in certain areas) [101,102,103,104]. Generally, head and neck spreading of IgG4-RD involve the two endocrine glands, thyroid, and pituitary gland, in addition to lacrimal, and salivary glands, orbit, as well as meningeal area [105]. Another classification of IgG4-RD includes the subgroup of conditions related to the nervous system (meningitis and hypophysitis), while peripheral neuropathy is exceptional [106].

### 4.4. New Roads for Riedel’s Thyroiditis

Overall, we identified nine single case reports (one patient with RT per paper, N = 9, female to male ratio of 2 to 1, aged between 34 and 79 years, 5/9 patients with serum IgG4 available data, 2/5 with high serum IgG4), four case series of patients with RT (more than one subject confirmed with RT per article, meaning a number of 2, 5, 6, and 8 persons, respectively, with a total of 21; female to male ratio was 4 to 2, 7 to 1, 4 to 1, and 1 to 1; 4/5 series provided data on IgG4 profile, but only three patients had serum IgG4 assays and 2/3 had abnormally high values), three studies in patients with IgG4-RD identified 25 patients with RT (one subject from a series of five cases, 23 individuals from a cohort of 628 subjects, and one individual from a retrospective of 12 persons diagnosed with retroperitoneal fibrosis), and two original studies specifically addressed IgG4-RTD components (one series of five subjects with IgG4-RTD identifying two cases of RT, and another study of 62 patients with post-thyroidectomy histological and immunohistochemistry report that detected nine patients with RT, a total N = 11, with IgG4 tissue profiling being available only for the first two mentioned patients in these studies).

A total of 66 subjects with RT are included in our analysis based on published cases. The rate of serum IgG4 evaluation remained low; normal values do not exclude RT [6,44,45,46,47,48,49,50,51,52,54,57,60,61,62,63].

It is possible that the frequent co-presence of autoimmune anti-thyroid antibodies in RT led us to focus our attention in the wrong direction (toward a particular type of autoimmune thyroiditis). RT should be assimilated to the larger picture of IgG4-RDs (or IgG4-associated sclerosing disease), which currently seems to display a chapter of its own in many medical and surgical domains other than thyroid. IgG4 immunostaining should become the new norm in RT. Applying the proposed criteria of IgG4-RTD might differentiate RT from other non-RT entities and raise the issue of potential non-thyroid IgG4-involvement in RT. This novel direction of classification and approach with respect to RT should emerge into an “omic” perspective of the condition, not just of a thyroid disorder, as part of IgG4-RD, with RT representing either a type of IgG4-RD with isolated organ involvement or being associated with synchronous/asynchronous multiple site spreading of IgG4-RD [1,32,33].

The new concept of IgG4-related RT is yet to be explored while gathering multi-level, multi-disciplinary data is mandatory. Routinely checking the thyroid status (TSH, free T4, and anti-thyroid blocking antibodies in association with thyroid ultrasound followed by a selection of patients to undergo core biopsy) in subjects confirmed with any type of IgG4-RD should be conducted, but the timing of serial check-ups is still an open issue. Suspecting RT should be associated with serum IgG4 testing (but the predictive value is uncertain so far) and a histological report and IgG4 stain since prompt glucocorticoid therapy might release local symptoms and avoid unnecessary complications in many cases. Further data on the IgG4-RT relationship are needed, as are interventional studies and longitudinal RT data.

## 5. Conclusions

The spectrum of IgG4-RTD varies from mild thyroid dysfunction (mostly hypothyroidism) in patients with high serum IgG4 and other non-thyroid manifestations (yet with a suggestive positive histological profile, particularly with a high amount of lymphoplasmacytic infiltration, especially IgG4expressing plasma cells) or with classical features of woody thyroiditis (RT) displaying a very aggressive profile and a poor outcome due to local evolution. The most recent 5-year RT analysis showed reports with a traditional RT (severe) presentation or cases with a fine index of suspicion concerning IgG4 contribution rather than through the clinical picture. The level of statistical evidence remains low in either scenario. Finally, from new pathogenic traits involving IgG4 to challenging endocrine and surgical aspects, RT remains one of the most interesting and fascinating topics in the thyroid domain.

## Figures and Tables

**Table 1:** Case and study sample analysis: inclusion and exclusion.

Inclusion Criteria	Exclusion Criteria
▪ Original papers (original studies, case series, and case reports)	▪ Experimental data, animal models
▪ Clinical data on humans	▪ Reviews, editorials
▪ Key search terms: “Riedel’s thyroiditis” (strategy 1) or “IgG4” AND “thyroid” (strategy 2)	▪ Types of thyroiditis other than Riedel’s thyroiditis
▪ Database: PubMed (+ free abstract access)	▪ Non-RT forms of IgG4-related thyroid disease (IgG4-related Hashimoto’s thyroiditis, fibrotic variant of Hashimoto’s thyroiditis, and IgG4-related Basedow–Graves’s disease)
▪ Full-length articles	
▪ Timeframe: 5-year analysis (starting in January 2019 until 15th May 2023)	

**Table 2:** Case reports involving the confirmation of RT according to our methods (the data start with the most recent, from 2023 to 2019) [6,44,45,46,47,48,49,50,51].

First Author Year of Publication Reference Number	Clinical Aspects Thyroid Profile	Management and Outcome
Pandev 2023 [6]	▪ Female aged 34 y ▪ Severe tracheal compression ▪ Bilateral RLN palsy ▪ Acute respiratory failure ▪ HypoT ▪ Positive Ab ▪ ↗CRP ▪ ↗ESR	▪ Tracheotomy (intraoperative pneumothorax)→tracheo-cutaneous fistula→late surgery for correction ▪ Open biopsy→RT confirmation ▪ Methylprednisolone ▪ Tamoxifen ▪ Mycophenolate mofetil (short course) ▪ T4 therapy (3 y of follow-up) for hypoT
Salhi 2023 [44]	▪ Female aged 48 y ▪ Rapid enlargement of pre-existing goiter + hypoT ▪ Local compression ▪ HypoPT ▪ ↗CRP	▪ T4 therapy for hypoT ▪ Calcium + alphacalcidol for hypoPT ▪ Decompression surgery + biopsy ▪ Good response to GC (hypoPT remission)
Er-Rahali 2021 [45]	▪ female aged 38 y ▪ 10-month history of hypoT ▪ Negative Ab ▪ Compressive symptoms ▪ Tracheal deviation and stenosis 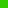 Serum IgG4 = 0.071 mg/dL (normal: 0.039–0.864)	▪ Non-conclusive FNA (twice) ▪ Subtotal thyroidectomy (started, but limited due to woody consistency) + temporary tracheostomy 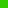 IgG4 antibodies + ve at IHC ▪ Post-operatory clinical progression ▪ GC therapy ▪ Transitory hypoPT requiring calcium + alfacalcidol ▪ Partial thrombosis at right internal jugular vein→3-month enoxaparin ▪ T4 therapy for hypoT
Góralska 2021 [46]	▪ Female aged 67 y ▪ Local pain (6-month evolution) ▪ ↗CRP ▪ HypoT corrected under LT4 (prior thyroidectomy) ▪ Positive Ab 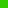 Serum IgG4 = 186 mg/dL (normal < 135)	▪ Non-conclusive FNA ▪ GC (refused surgery) ▪ T4 therapy for hypoT
Pacella 2021 [47]	▪ Male aged 53 y ▪ Retroperitoneal fibrosis diagnosed 4 y after RT (current normal IgG4)	▪ Obstructive uropathy→GC + stent placement ▪ For RT: the patient had decompression thyroid surgery 4 y before
Navarro-Sánchez 2020 [48]	▪ Male aged 69 y ▪ 7-month history of dysphagia + hypoT ▪ Positive Ab ▪ ↗CRP 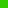 Serum IgG4 = 621 mg/dL (normal:3–201)	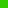 Biopsy→IHC: IgG4/IgG = 40% ▪ T4 therapy for hypoT ▪ Tamoxifen (20 mg/day, 2 months)
Shafi 2020 [49]	▪ Male aged 35 y ▪ Thyroid enlargement (15 cm) ▪ HypoT, hypoPT ▪ Positive Ab ▪ ↗CRP 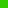 Normal serum IgG4	▪ Non-conclusive FNA ▪ Partial thyroidectomy ▪ Intra-operatory biopsy ▪ T4 therapy for hypoT ▪ Calcium + alfacalcidol (2 y) for hypoPT ▪ GC (prednisolone 5 mg/day, 2 y) ▪ Tamoxifen (20 mg/day, 3 months)→bilateral internal jugular vein and intracerebral venous thrombosis→enoxaparin (3 months)→rivaroxaban 20 mg/day (2 y) ▪ Rituximab not tolerated
Mammen 2019 [50]	▪ Female aged 51 y ▪ History of HT ▪ Presentation as multinodular goiter ▪ HypoT ▪ Compressive symptoms 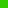 Serum IgG4 = 8 mg/dL (normal:1–291)	▪ At onset: methylprednisolone (125 mg every 6 h, 2 days) ▪ Planned thyroidectomy but aborted ▪ Intra-operatory biopsy ▪ Prednisone (60 mg per day maximum dose) + tamoxifen (30 mg twice per day, 1 y) ▪ →disease progression: IV rituximab (375 mg/m^2^ every 3 weeks, 4 doses)→stop tamoxifen, ↘ GC dose
Kumar 2019 [51]	▪ Male aged 79 y ▪ 2 y history of compressive symptoms	▪ Unsuccessful FNA (twice) ▪ Diagnosis based on needle core biopsy ▪ GC (follow-up for 2 months→loss of evidence)

Abbreviations: Ab = thyroid antibodies (anti-thyroid peroxidase and anti-thyroglobulin antibodies); CRP = C-reactive protein; ESR = erythrocyte sedimentation rate; FNA = fine needle aspiration; GC = glucocorticoid therapy; IV = intravenous; IHC = immunohistochemistry; HT = Hashimoto’s thyroiditis; hypoT = hypothyroidism; hypoPT = hypoparathyroidism; HPF = high-power field; RLN = recurrent laryngeal nerve; RT = Riedel’s thyroiditis; T4 = thyroxine; and y = year (green points represent the data we have concerning the IgG4 profile).

**Table 3:** Case series specifically addressing RT (more than 1 patient with RT per study); the data start with the most recent publication [52,54,57,60].

First Author Year of Publication Reference Number	Clinical Aspects Thyroid Profile	Management and Outcome
Sadacharan 2023 [54]	▪ N = 6 patients with RT (female/male = 4/2) ▪ Onset with compressive symptoms	▪ 4/6 patients—total thyroidectomies ▪ 1/6 patients—hemithyroidectomy ▪ 1/6 patient—GC
Gökçay Canpolat 2021 [52]	▪ N = 8 patients with RT (F/M = 7/1) ▪ 2/8 patients with positive Ab 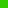 Negative serum IgG4 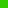 IHC report: IgG4/IgG >40% (2/8)	▪ GC: ▪ Severe presentation: IV prednisolone 2–3 mg/kg/day, 2–7 days ▪ Oral: 0.8–1 mg/kg/day, 4–8 weeks ▪ Overall: 6–12 months depending on the severity ▪ Tamoxifen (10–20 mg/day): ▪ 1–3 months (before GC is withdrawn) ▪ Azathioprine + colchicine (1 patient with polyserositis) ▪ Periodic check-up: every 3–6 months ▪ Median follow-up: 67 months
Yu 2021 [60]	▪ N = 5 patients with RT (F/M = 4/1) 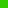 Serum IgG4 (2/5p): of 167, respective 22 g/L (normal: 0.03–2.01) IHC report: 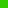 IgG4–positive plasma cells/HPF of 80,8,50,10,22% 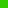 IgG4/IgG ratio of 76,80,43,19,28% 3/5 *p* with +ve Ab	▪ 1 patient with retroperitoneal fibrosis ▪ 2/5 patients with thyroidectomy ▪ 3/5 patients with GC
Blanco 2019 [57]	▪ N = 2 patients with RT Case 1: female aged 38 y 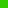 IHC: positive IgG4 Case 2: male aged 56 y IHC report: 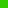 IgG4-positive plasma cells/HPF of 10 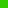 IgG4/IgG ratio >40%	▪ Total thyroidectomy (both) ▪ Non-conclusive FNA (female case) ▪ T4 therapy for hypoT at presentation (male case)

Abbreviations: Ab = antibodies; C = case; F = female; GC = glucocorticoids; IV = intravenous; Ig = immunoglobulin; IHC = immunohistochemistry; Ig = immunoglobulin; N = number of patients; and RT = Riedel’s thyroiditis (green points represent the data we have concerning the IgG4 profile).

**Table 4:** Studies with patients diagnosed with different forms of IgG4-related disease: thyroid findings (the data start from 2023) [61,62,63].

First Author Publication Year Reference Number	Study Design Studied Population	IgG4-Related Thyroid Findings
Nandi 2023 [61]	▪ Case series ▪ N = 5 patients with cardiovascular findings (aortitis/peri-aortitis) in IgG4-RD	▪ 1/5 patients with RT (biopsy confirmation) ▪ Normal thyroid function ▪ Biopsy confirmation ▪ Glucocorticoid therapy 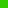 Serum IgG4 = 3.32 g/L (normal: 0.03–2.01)
Sun 2023 [62]	▪ Retrospective cohort study ▪ N = 628 patients with IgG4 ▪ N′ = 93 patients with positive family history of autoimmune diseases ▪ N″ = 535 patients with negative family history of autoimmune diseases	▪ RT prevalence: N′ > N″ = 10.9% (N = 10 patients) versus 2.4% (N = 13), *p* = 0.001
Azizi 2020 [63]	▪ Retrospective study ▪ N = 12 patients with retroperitoneal fibrosis	▪ Prevalence: 1/12 patients

Abbreviations: Ig = immunoglobulin; RT = Riedel’s thyroiditis; and N = number of patients (green points represent the data we have concerning the IgG4 profile).

**Table 5:** Studies addressing the components of IgG4-RTD, including RT [53,77].

First Author Year of Publication Reference Number	Clinical Aspects Thyroid Profile	Management and Outcome
Jin 2022 [53]	▪ N = 5 patients with IgG4-RTD ▪ N1 = 2 patients with IgG4-related RT ▪ Case 1: male aged 41 y ▪ 6-month history of hypoT under T4 therapy ▪ Positive Ab ▪ Case 2: male aged 72 y ▪ Incidental thyroid nodule (3.4 cm) ▪ Transitory (4-month) thyrotoxicosis ▪ Nno hypoT	▪ Case 1: total thyroidectomy (pre-operatory CNB results: papillary carcinoma) Post-operatory IHC: 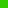 IgG4 plasma cells/HPF = 93 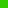 IgG4/IgG ratio = 42% ▪ Case 2: CNB results 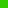 IgG4 plasma cells/HPF = 75 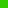 IgG4/IgG ratio = 37% 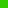 Serum IgG4 = 91 mg/dL
Gvianishvili 2019 [77]	▪ N = 62 patients with post-thyroidectomy histological positive IHC reports ▪ N1 = 9 patients with RT	▪ RT: moderate positive TTF-1, S100 protein, negative malignant markers CD56, p63

Abbreviations: Ab = anti-thyroid antibodies; CNB = core needle biopsy; IHC = immunohistochemistry; IgG4-RTD = immunoglobulin G4-related thyroid disease, hypoT = hypothyroidism; and RT = Riedel’s thyroiditis (green points represent the data we have concerning the IgG4 profile).

## Data Availability

Not applicable.

## Abbreviations

ACR	American College of Rheumatology
CRP	C-reactive protein
ESR	Erythrocyte sedimentation rate
EULAR	European League Against Rheumatism
HPF	High-power field
Ig	Immunoglobulin
IgG4-RD	Immunoglobulin IG4-related disease
IgG4-RTD	IgG4-related thyroid disease
RT	Riedel’s thyroiditis
TGF-*β*	Transforming growth factor beta
Tc	Technetium
